# A qualitative assessment of facility readiness and barriers to the facility-based management of childhood severe acute malnutrition in the public healthcare settings in Bangladesh

**DOI:** 10.1017/S1368980022002014

**Published:** 2022-11

**Authors:** Shah Mohammad Fahim, Md Ridwan Islam, Md Golam Rasul, Mohammad Jyoti Raihan, Nafi Mohammad Ali, Md Mofijul Islam Bulbul, Tahmeed Ahmed

**Affiliations:** 1 Nutrition and Clinical Services Division (NCSD), International Centre for Diarrhoeal Disease Research, Bangladesh (icddr,b), 68, Shaheed Tajuddin Ahmed Sarani, Mohakhali, Dhaka 1212, Bangladesh; 2 National Nutrition Services, Institute of Public Health Nutrition, Ministry of Health and Family Welfare, Government of Bangladesh, Dhaka, Bangladesh; 3 Office of the Executive Director, International Centre for Diarrhoeal Disease Research, Bangladesh (icddr,b), Dhaka, Bangladesh; 4 Department of Global Health, University of Washington, Seattle, Washington, USA; 5 Department of Public Health Nutrition, James P Grant School of Public Health, BRAC University, Dhaka, Bangladesh

**Keywords:** Severe acute malnutrition, Under-five children, SAM facility-based management, Facility readiness, Barriers

## Abstract

**Objective:**

To assess facility readiness and identify barriers to the facility-based management of childhood severe acute malnutrition (SAM) in public healthcare settings.

**Design:**

Qualitative methods were applied to assess readiness and identify different perspectives on barriers to the facility-based management of children with SAM. Data collection was done using in-depth interviews, key informant interviews, exit interviews and pre-tested observation tools.

**Settings:**

Two tertiary care and four district hospitals in Rangpur and Sylhet Divisions of Bangladesh.

**Participants:**

Healthcare professionals and caregivers of children with SAM.

**Results:**

Anthropometric tools, glucometer, medicines, F-75, F-100 and national guidelines for facility-based management of childhood SAM were found unavailable in some of the hospitals. Sitting and sleeping arrangements for the caregivers were absent in all of the chosen facilities. We identified a combination of health system and contextual barriers that inhibited the facility-based management of SAM. The health system barriers include inadequate manpower, rapid turnover of staff, increased workload, lack of training and lack of adherence to management protocol. The major facility barriers were insufficient space and unavailability of required equipment, medicines and foods for hospitalised children with SAM. The reluctance of caregivers to complete the treatment regimen, their insufficient knowledge regarding proper feeding, increased number of attendants and poverty of parents were the principal contextual barriers.

**Conclusions:**

The study findings provide insights on barriers that are curbing the facility-based management of SAM and emphasise policy efforts to develop feasible interventions to reduce the barriers and ensure the preparedness of the facilities for effective service delivery.

Severe acute malnutrition (SAM) affects approximately 14·3 million under-five children globally^(1)^. This serious form of undernutrition, defined as a mid-upper arm circumference < 11·5 cm or a weight-for-height *Z*-score < − 3 with or without oedema, is attributable to numerous life-threatening conditions during the early years of life^(2–4)^. The condition is often multifactorial and characterised by a number of clinical manifestations including muscle wasting and nutritional oedema^(3)^. SAM was also found to be associated with co-infections, inflammation and enteropathy^(3)^. This deadly form of acute malnutrition may result in deceleration of immune functions, increased risk of infectious diseases and ultimately accelerates mortality, particularly in under-five children^(5)^. SAM is considered to be one of the leading causes of childhood deaths in hospital settings^(6)^. The ongoing pandemic of COVID-19 further deteriorated the condition causing an increased rate of morbidity and mortality in severely malnourished children warranting immediate attention and utmost care to reduce the deaths from SAM in young children. However, the lack of effective healthcare services is a serious concern for the management of SAM in resource limited settings^(7)^. The WHO has developed guidelines for inpatient management of children with SAM in order to reduce the unfavourable outcomes as well as mortality^(8)^. The mortality from severe malnutrition can be reduced substantially if the management guidelines are precisely followed by the healthcare providers^(9,10)^.

Due to inappropriate application of these guidelines, the mortality from severe wasting remains persistently high in many low- and middle-income countries^(11)^. SAM accounts for approximately one to two million under-five child deaths/year globally^(12,13)^. The number of severely wasted children is considerably high in South Asian countries, including Bangladesh^(1)^. As per Bangladesh Demographic and Health Survey 2017–2018, the prevalence of SAM was estimated 2 % among children aged 6–59 months which accounts for more than 0·3 million under-five children^(1,14)^. The country has adopted national guidelines for successful management of childhood SAM to avert its adverse consequences^(15,16)^. However, the outcome of inpatient care for children with SAM is not yet up to the mark^(11,17)^. There may have potential barriers within the health systems, both on demand and supply sides, which are primarily responsible for such undesirable outcomes in relation to facility-based management of SAM in children. Evidence suggests that facility-based management of SAM can be affected by multiple factors^(18–20)^. Besides, the readiness of a facility to deliver standard healthcare services in order to manage critically ill patients with SAM is also crucial to ensure proper facility-based management^(21,22)^. To that end, it is imperative to assess the readiness in management of childhood SAM in the public healthcare facilities of Bangladesh. In addition, identification of potential barriers would help to design effective policies to ensure appropriate inpatient management of children with SAM. Therefore, we sought to assess readiness of the facilities and identify barriers to the facility-based management of childhood SAM in public healthcare settings in Bangladesh.

## Methods

### Study design

A qualitative study was done to assess the facility readiness as well as different perspectives on barriers and challenges to the facility-based management of children with SAM. Data were collected using in-depth interviews (IDI), key informant interviews, exit interviews and a pre-tested observation tool. IDI were conducted among doctors and nurses who were directly involved in the inpatient management of children with SAM, KII were conducted among key personnel who were considered as experts in the facility-based management of SAM and EI were conducted among caregivers of the patients with SAM. The interview guidelines and observation checklist were prepared based on the national guidelines and findings from previous studies. We did pre-test the interview guidelines after interviewing ten participants (six for IDI, two for key informant interviews, two for exit interviews) in a tertiary care hospital. The observation checklist was also pre-tested in the paediatric ward and nutrition rehabilitation unit of a tertiary care hospital. Results from IDI and KII were recorded and analysed to identify the barriers from the perspective of supply side. Information from EI were audiotaped and analysed to point out the barriers from demand side. The observation checklist was developed and pre-tested with the purpose of methodological triangulation of data obtained by different techniques.

### Study settings and participants

The study locations as well as the respondents for IDI and KII were selected purposively. First, two administrative divisions of Bangladesh were selected based on the prevalence of wasting: Rangpur (with lowest prevalence, 7·3 %) and Sylhet (with highest prevalence, 10·4 %)^(14)^. Subsequently, one tertiary care teaching facility and two district hospitals were chosen from each division. The teaching hospitals were Sylhet MAG Osmani Medical College and Hospital (SOMCH) and Rangpur Medical College and Hospital (RMCH). These two institutions are the largest and oldest healthcare facility in Sylhet and Rangpur divisions. There are fully functional SAM units or paediatric wards to provide inpatient services to the children with SAM. Moreover, these are the hospitals with highest number of under-five children admissions in the past year in their respective divisions.

For the district hospital, we did select Habiganj District Hospital and Moulvibazar District Hospital from the Sylhet division and Thakurgaon District Hospital and Kurigram District Hospital from the Rangpur division. All these facilities had highest number of under-five children admissions in the previous years compared with other district hospitals in their respective divisions^(23)^. Additionally, there present functional SAM wards to provide management to undernourished children as per the national guidelines. Overall, two tertiary care teaching facilities and four secondary care district hospitals were selected where severely malnourished children get admitted for inpatient care according to the national guidelines^(16)^.

Finally, healthcare providers, both doctors and nurses posted in paediatric wards, were selected from the chosen healthcare facilities as the respondents for IDI. We did only interview doctors and nurses for IDI if they were directly involved in the facility-based management of children with SAM. The key informants were nominated based on their position as well as expertise on the facility-based management of SAM. Professors, Head of the Departments or Hospital Supervisors were primarily considered for key informant interviews. In addition, we interviewed Line Director and Program Manager of National Nutrition Services as key informants as National Nutrition Services is the public authority to launch and oversee nutrition programmes in the country. Exit interviews (exit interviews) were done with the caregivers of hospitalised children with SAM. We found only four hospitalised children with SAM during our visit to the selected hospitals. Therefore, the number of EI was four.

### Definition and components of facility readiness

Facility readiness was defined as the overall capacity of the facility to deliver optimum healthcare services to the children with SAM^(24)^. It was measured by the availability of tracer items in six domains: (1) instruments for SAM identification; (2) documents related to management of SAM; (3) necessary medical equipment and devices; (4) diagnostic facilities and essential medicines; (5) amenities to maintain standard sanitation and hygiene practices and (6) overall structure and condition of the ward^(24,25)^.

### Data collection

The qualitative data collection was carried out between September 2020 and January 2021. Overall, twenty-six IDI, thirteen KII and four EI were conducted until the saturation of data. In addition, 28 h of observation was done in each of the selected hospitals to monitor ward infrastructure, facility preparedness and SAM management practices by the healthcare professionals. Observation data were collected between 8 am to 10 pm for two consecutive days. In Bangladesh, hospital duties are divided into three shifts: morning (8 am to 3 pm), evening (3 pm to 10 pm) and night (10 pm to 8 am). The observation data were collected in morning and evening shifts of the hospital duties. We primarily investigated facility-based SAM management barriers and barriers from the supply side through IDI. We also explored facility related barriers, challenges related to possible solutions and recommendations by KII and demand side barriers and challenges through EI. The interviews as well as observation were facilitated by the study investigators.

### Data analysis

All the interviews were conducted by trained and experienced interviewers and recorded accordingly in Bengali language. The interview team consists of physicians and anthropologists who received training regarding inpatient management of SAM and have experience of conducting qualitative interviews in the hospital settings. The audio recordings were transcribed manually in Bengali. Transcribed data were described and analysed using qualitative content analysis method^(26)^. Subsequently, a research team with expertise in treating children with SAM, public health and anthropology conducted thematic analysis by manual coding where each theme portrayed barriers from either supply or demand sides. A combined theoretical and inductive coding approach was used by the members where they each went through all of the transcripts and generated codes. Potential codes and themes were reviewed and revised among all the members until agreement was reached. Finally, the relevant quotes were translated to English from Bengali. The investigators translated the quotes by themselves. However, there was no standard protocol that has been followed for translation. Observation data collected from the facilities during the study were analysed based on the WHO manual on Service Availability and Readiness Assessment (SARA Tool)^(24)^. Two physicians and one anthropologist collected the observation data using a pre-tested semi-structured observation checklist. They observed the paediatric wards including SAM corners and their associated parts. SAM corner is a separate space within the paediatric ward dedicated only for the children diagnosed with SAM. Such corners are established in all the facilities where inpatient management of children with SAM are regulated. The observation was done for 28 h in two consecutive days including the morning and evening shifts extending from 8 am to 10 pm. The night shift was excluded because most of the management is usually covered within morning and evening shifts. Under ‘broad spectrum antibiotics’, we searched for at least one of these three injectable drugs; ampicillin, gentamycin and/or ceftriaxone to be available during our visit to the wards. In ‘essential micronutrients’ category, we checked for Zn, multivitamins, folic acid, vitamin A, potassium and magnesium supplementation collectively. We have recorded it as ‘absent’ if any of these was found missing in the selected facilities. With regard to ‘laboratory investigation facilities’, we looked at whether routine blood examinations including complete blood count, serum electrolytes and blood cultures were carried out in the facilities. No radiological or sonographic investigations were included in our observation checklist. Facilities that did not fulfill all the criteria to be deemed prepared were defined as ‘not ready’ and vice versa.

### Reliability and validity

In this study, we interviewed the participants until data saturation to ensure the reliability of information regarding barriers and challenges to the facility-based management of childhood SAM. Different methods can be used in qualitative research to ensure data validity and reliability. Among them triangulation (triangulation among different interview and observation tools, methods and researchers), member checking and peer debriefing are prominent^(27)^. In our study, we have triangulated our data with different research findings. Data from interviews and observations were also triangulated in our analysis. In addition, peer debriefing was done regularly to ensure data reliability and saturation. Internal validity was ascertained by triangulating themes among interviews with different types of respondents (i.e. healthcare providers, subject experts and caregivers of the children) and observation of facility readiness as well as management practices. Validity of the findings was reinforced by the application of qualitative methods and reporting the perceptions as stated by the respondents in their own words.

## Results

### Participant characteristics

A total of forty-three participants were interviewed (twenty-one doctors, eighteen nurses and four caregivers) from six hospitals. The socio-demographic characteristics of the interviewees attended IDI and key informant interviews are reported in Table 1. A majority of the participants were aged between 31 and 40 years. Among the IDI participants, seven (26·9 %) received training on facility-based management of SAM. Out of the four respondents of exit interviews, three were female.

**Table 1:** Summary of socio-demographic features of participants

Variables	Categories	IDI (*n* 26)	KII (*n* 13)
Age	≤30 years	6	0
	31–40 years	13	1
	41–50 years	4	6
	>50 years	3	6
Sex	Male	7	6
	Female	19	7
Education	Diploma	12	6
	Graduation	14	7
Occupation	Doctor	14	7
	Nurse	12	6
Designation/Role	Line director, NNS	–	1
	Deputy programme manager, NNS	–	1
	Deputy civil surgeon	–	1
	Professor	–	1
	Superintendent	–	1
	Consultant	3	2
	Registrar	3	–
	Assistant registrar	3	–
	Medical officer	5	–
	Senior staff nurse	12	–
	Nursing supervisor/nursing in-charge	–	6
Type of facilities	Secondary care hospital	14	7
	Tertiary care hospital	12	4
	NNS	–	2
Training on facility-based management of SAM	Received	7	–
	Not received	19	–

NNS, national nutrition services; SAM, severe acute malnutrition.

### Facility readiness for management of children with severe acute malnutrition

A total of 168 h of observation was conducted in the selected hospitals during the project period. Anthropometric tools, for instance, length board and mid-upper arm circumference tape, were unavailable in three (50 %) out of six facilities. However, paediatric weighing scales were present in all the selected facilities. Glucometer, an important tool to assess hypoglycaemia, was not available in two out of six hospitals. Posters on nutrition education were present in four facilities. However, the national guidelines for facility-based management of SAM were found only in two of the selected hospitals. Sitting or sleeping arrangements for the caregivers were not present in any of the chosen hospitals. The caregivers and attendants took rest on the same bed that the children were kept on or lay down on the floor. In addition, the general condition and cleanliness of the wards and toilets were found below standard that means these were not properly cleaned. Moreover, the sanitary conditions were found unhygienic. In each facility, caregivers had to buy drinking water from outside due to lack of drinkable water sources in the hospitals. Essential laboratory investigation facilities were available only in one hospital. Moreover, supplies of essential equipment, medicines and foods were not available in some of the selected hospitals. Only two of the selected hospitals had stock of essential micronutrients, while ReSoMal was not present in any of the facilities. Findings on readiness of facilities and availability of essential equipment to ensure treatment services are described in Table 2. All the facilities were seemed to be ‘not ready’ as none of them could fulfill the required criteria to be deemed as ready for providing effective services to children with SAM.

**Table 2:** Facility readiness for management of children with SAM based on data obtained from observation

Variables	Categories	Rangpur division		Sylhet division	
		District hospitals (*n* 2)	Medical College hospital (*n* 1)	District hospitals (*n* 2)	Medical College hospital (*n* 1)
Instruments for SAM identification					
Weighing scale	Present	2	1	2	1
Length board	Present	1	0	2	0
MUAC tape	Present	2	0	1	0
Documents related to management of SAM					
Growth charts	Present	2	1	2	1
National guidelines for facility-based management of SAM	Present	1	0	1	0
Picture cards/posters on nutrition education	Present	1	1	1	1
Record keeping register/reporting forms	Present	2	1	2	1
Medical equipment or devices required for management of SAM					
Glucometer	Present	2	1	1	0
Thermometer	Present	1	1	1	0
Cotton	Present	2	1	2	1
Gauge	Present	2	1	2	1
Micropore	Present	1	1	1	1
Nasogastric tube	Present	0	1	0	1
Cannula	Present	1	1	2	1
Syringe	Present	2	1	2	1
Chlorhexidine Gluconate & Isopropyl alcohol	Present	0	1	1	1
Gloves	Present	1	1	2	1
Apron	Present	2	1	2	1
Stethoscope	Present	2	1	2	1
Pediatric sphygmomanometer	Present	1	1	1	0
Diagnostic capacity and essential medicines					
Availability of essential laboratory investigation facilities	Present	0	1	0	0
F-75	Present	0	0	2	1
F-100	Present	1	0	1	1
Broad-spectrum antibiotics	Present	2	1	2	0
Essential micronutrients	Present	1	0	1	0
ORS	Present	2	1	2	1
ReSoMal	Present	0	0	0	0
Sanitation and hygiene					
Bedsheet	Clean	0	0	1	0
Floor	Clean	0	1	1	0
Floor cleaning material	Present	2	1	2	1
Condition of toilet	Clean	1	1	0	0
Source of pure drinking water	Present	0	0	0	0
Soap	Present	1	0	2	1
Towel	Present	0	1	1	1
Structure and condition of ward					
Sitting arrangements of the attendant	Present	0	0	0	0
Sleeping arrangement of the attendant	Present	0	0	0	0
Medicine shelf	Present	2	1	2	1
Fan	Adequate	2	1	2	0
Light	Adequate	2	1	2	1
Uninterrupted electricity supply (Generators/IPS)	Present	1	1	2	1

SAM, severe acute malnutrition; MUAC, mid-upper arm circumference; ReSoMal, rehydration solution for malnutrition; IPS, instant power supply.

### Barriers to the facility-based management of childhood severe acute malnutrition

A total of forty-three interviews were conducted in the selected facilities that assisted us in identifying the barriers to the facility-based management of SAM in public healthcare settings. Barriers related to health system, hospital settings and demand side were detected after analysis of the interview findings. Table 3 describes the specific barriers under each domain with significant quotes from the respondents.

**Table 3:** Barriers to the facility-based management of childhood SAM

Domain	Specific barriers	Illustrative quotes
Health system barriers	Inadequate manpower	‘We have shortage of space, and we do not have enough staff to ensure proper services. We are less in number to perform our duties in the ward. On an average, we have to look after more than fifteen or sixteen patients per head/d. It is really difficult for a single person to deal with such a huge number of patients. Sometimes the number of patients increases much more. For example, today there are one hundred and seventy-four patients in the ward where we are only three staff who are present in the morning shift. Just calculate how many patients per head?’ (IDI-Participant 26)
	Rapid turnover of staff	‘The doctors and nurses have to play a huge role in the management of patients with SAM. We have an ongoing training process. The healthcare staff are receiving that training. Sometimes trainers from IPHN visit to different places and train the healthcare staff. But, those who received the training have left their designated workplace, as they were transferred elsewhere. This kind of turnover is a challenge for us. The rapid turnover is more common for doctors. However, nurses are also leaving but on a lesser scale.’ (KII-Participant 12)
	Increased workload	‘In addition to SCANU (a ward name) and general pediatrics ward, I am also responsible for working in the gynae ward. So, currently I work for three different wards with an increased workload.’ (IDI-Participant 21)
	Lack of training	‘Actually, we need trained nurses because at present we are providing services being in dark. Yes, we did complete our BSc or diploma degree in Nursing, where we learned about our duties. But the things you guys are talking about, for example, steps of management, national guidelines, were not that much covered in our curriculum. We could have provided better services if we had training on these topics.’ (IDI-Participant 2)
	Lack of adherence to management protocol as recommended in the national guidelines	‘In case of hypothermia, we usually put the child in the warmer if he/she is small in size. For the other cases, we wrap the baby properly with clothes and ensure regular feeding. Sometimes we administer dexamethasone (steroid) to the patients with hypothermia. I did administer it a lot of the time if the child was found to be in a critical condition.’ (IDI-Participant 14)
Facility barriers	Unavailability of functional anthropometry tools	‘Even if we miss the weight of a normal child, we can’t miss it for a child with SAM. We need to measure weight and height regularly to ensure proper diagnosis. It is also imperative to observe other anthropometric parameters for these children. So, it would be better if we could ensure regular supply of these tools in the hospitals. Some anthropometric tools can only be assigned for the children with SAM. For example, it would be helpful if we had a weight and a length machine, a warmer, and adequate supplies of MUAC tapes in the ward. Although we have supply of some of these things, but sometimes it seems to be difficult to manage these tools in the facility. However, we should not feel ashamed to disclose about the lacking of these items in the ward.’ (IDI-Participant 5) ‘We only measure weight. We only have weight machine here.’ (IDI-participant 13)
	Unavailability of national guidelines in the ward	‘No, we don’t have the guidelines in our ward.’ (IDI-Participant 26)
	Unavailability of essential medicines	‘Most of the time, ten percent glucose is not supplied by the authority which is needed to treat hypoglycemia. In that case, we have to write down the name of medicine in a slip and give it to the patient’s attendant to collect the medicine from outside. They visit to the pharmacy located outside the hospital and buy the medicine. We can only administer the drug when they bring it from outside. This is an obstacle to deliver prompt service to the patients.’ (KII-Participant 6)
	Unavailability of supplies and equipment	‘We only get Zinc syrup from the hospital. We don’t have supply of potassium, magnesium, retinal vitamin capsule, and multivitamin supplements. So, the patient has to buy all these medicines from the outside. Ampicillin and gentamycin are available, but these antibiotics are not enough to manage the patients who usually get admitted here as most of them come with a more complicated condition. We also have supplies of ceftriaxone antibiotic. But if we want to prescribe any other antibiotics, they have to buy those from outside. The hospital doesn’t offer anything related to blood transfusion. We need a blood bag, blood grouping test, Rh typing test, and complete screening of the donor. However, we don’t receive anything related to these things from the hospital. The patient has to manage everything by themselves. They have to bear all of these costs. We don’t receive any ointment from the hospital for dermatosis. It is also applicable for eyedrops required for the children suffering from corneal ulceration. So, we can’t administer these drugs until they buy it by themselves. The patient has to buy almost everything from outside for the treatment.’ (IDI-Participant 15) ‘We can’t even conduct the first step of management properly. We don’t have a warmer to prevent and manage hypothermia. We don’t even have any glucometer to prevent or treat hypoglycemia.’ (IDI-Participant 15)
	Difficulties in managing foods for admitted children	‘The main problem we face is related to diet. If we had regular supplies of F-75, it would have been easier for us to manage these patients. Because the sisters (nurses) do not prepare the food. The attendant of the patient has to prepare it for the admitted child. The attendants can’t always follow our instructions to prepare the food properly. Although they follow the instructions at the beginning, but later on they feed the child on their own accord.’ (IDI-Participant 3)
	Unavailability of resources for essential laboratory investigations	‘We can’t do any diagnostic tests in our facility. We could understand what tests are needed to be done. But most of the time we have to diagnose clinically. We can see the children are recovering, but we are not doing something ethical or scientifically sound because we were supposed to conduct tests to confirm the decision. It would have been better if we could diagnose based on appropriate clinical tests. We have two major problems: one is scarcity of manpower and the other is lack of adequate investigation facilities.’ (IDI-Participant 14)
	Insufficient space and beds in the SAM corner	‘We have limited number of beds in the SAM corner. There are only two beds. If we had ten to twelve beds, it would have been ideal. In that case, the distance between the beds would have been better and we could have provided better services.’ (IDI-Participant 7) ‘Most of the time we can’t provide beds to patients. We have to accommodate two or three patients in a single bed. Patients don’t want to stay in the hospital due to this problem. It is very common that they leave or become absconded before completing the treatment.’ (IDI- Participant 4)
Demand-side barriers	Increased number of attendants affecting services	‘There are four, five, or six attendants with only one patient. The attendants come here by CNGs (a transport vehicle in Bangladesh) which are fully packed with them. They don’t try to understand the problem of overcrowding in the ward. Most of them don’t wear a mask in this corona situation. Even if we ask them nicely, they don’t listen to us. They do smoke, drink tea, and eat lunch in the same ward and create havoc. We can’t maintain optimum treatment protocol due to the overcrowding problem.’ (IDI-participant 24)
	Insufficient knowledge of the caregivers on malnutrition and proper feeding	‘The caregivers do not feed the child properly. Sometimes they feed the child shuji. They don’t offer breastmilk to the child, rather they feed the baby with other kinds of milk. They don’t even prepare the other milk solutions properly. It is very common that they don’t use the correct amount of water in the solution. So, the baby turns to SAM due to inappropriate feeding.’ (IDI-participant 22)
	Lack of motivation and compliance to complete the treatment regimen by caregivers	‘We have to face many difficulties while working in the hospital. Mainly, we have to counsel the patient parties a lot. Especially, the SAM patients don’t want to stay for a long time. The caregivers don’t have the patience to complete the treatment. The mothers get annoyed. But we try to keep them here by counseling.’ (IDI-Participant 7) ‘The main problem we face is that the patients leave the hospital before completing the treatment. After staying for two or three days, they decide not to stay at hospital anymore. Then they leave or abscond.’ (KII-Participant 10)
	Getting admitted only when the patient develops serious complications as parents are not motivated to seek early help	‘Most of the children with SAM come to us in a critically ill condition. We often observe that these children also have other diagnoses, for example, pneumonia, septicemia, or heart failure.’ (KII-Participant 6) ‘These patients report with severe septicemia or other problems like shock. These types of cases are challenging ever after reversal. That is why they even expire sometimes.’ (KII-Participant 9)
	Poverty of parents	‘These children usually belong to ultra-poor families. The family members don’t realize that their child is lean and thin, and he/she can die from this illness. They are even unaware of the future consequences if the child survives. They keep saying that their cattle at home are unattended, so they have to leave early from the hospital.’ (KII-Participant 12)

#### Health system barriers

A number of health system-related barriers were identified in this study that are highlighted with quotes in Table 3. Insufficient manpower resulting in increased workload, lack of training, rapid turnover of healthcare staff and healthcare professionals’ lack of adherence to the guideline suggestions were found as key challenges in ensuring the proper management of SAM. The quotes from the study respondents clearly emphasised on requirement of training for healthcare providers. We found that only seven out of twenty-six26 IDI participants had received training on inpatient management of SAM. Lack of adequate manpower in the wards was an important barrier recalled in several interviews. Our observation also supports the shortage of service providers in the selected facilities. We observed that healthcare providers rely more on their own judgement rather than the protocol outlined in the national guidelines. As reported in Table 3, a healthcare provider stated that he administers dexamethasone in children with hypothermia. However, prescribing steroids to such patients is not recommended in the management guidelines. We found only four out of twelve nurses (33·3 %) who were aware of the national guidelines as well as the steps to manage children with SAM. This can be considered as a potential barrier from the service delivery side. In addition, rapid turnover of staff was found as another obstacle in managing severely malnourished children. Rapid turnover denotes to frequent posting of healthcare staff from one facility to other facilities. A number of interviewees stated that such turnover initiate difficulties in the management of children with SAM because it takes time to make the new staff oriented with the guidelines for facility-based management of SAM.

#### Facility-related barriers

Facility-related barriers were identified by analysing the interviews and triangulating the interview findings with observation data. The unavailability of functional anthropometry tools was one of the major facility-related barriers. Length boards and mid-upper arm circumference tapes were found only in three of the six selected hospitals. Although weighing machines were available in all the selected facilities, the scales were not readily available in the paediatric wards. Some of the respondents mentioned that sometimes they face difficulties to measure weight due to inadequacy of the weighing scales in the wards. Nevertheless, they never calculate *Z* scores as length is not measured regularly in the wards. In that case, SAM identification is done based on the visual inspection of muscle wasting or oedema. Inadequacy of medicines, insufficient treatment supplies and unavailability of resources for laboratory investigations were also detected as major obstacles from the interviews. These findings remain analogous when triangulated with inspection data. We observed that attendants had to buy medicines and supplies due to unavailability of these items in the hospitals. We also observed similar findings in terms of laboratory investigations. Besides, unavailability of the national guidelines in the paediatric wards, difficulties in managing foods for admitted children and insufficient space and beds in the SAM corner were found as barriers from the supply side affecting the inpatient care.

#### Demand-side barriers

Important themes that emerged to identify barriers from the service recipient side were increased number of attendants affecting services, the financial crisis of parents, insufficient knowledge of the caregivers regarding malnutrition and proper feeding practices and their reluctance to complete the treatment regimen. Apart from these, parents not seeking early help was also recognised as a barrier from the demand side. From a quote related to an increased number of attendants in the ward (Table 3), it is conspicuous that the service providers were discontented regarding the issue. It was affecting service delivery severely. In addition, a large number of attendants were not wearing masks putting the healthcare providers at risk of being infected with SARS-CoV-2. According to the quote in Table 3, the lack of knowledge of caregivers regarding malnutrition affected the child’s recovery. Incompletion of treatment at the hospitals was also a serious issue that was raised by the interviewees.

Exit interviews were conducted to discover potential barriers that are being faced by the caregivers in the process of receiving treatment services. They received basic information about nutrition and food preparation techniques from the healthcare providers upon admission to the hospitals. Dissatisfaction regarding general conditions and cleanliness of the paediatric wards was one of the main concerns raised by the caregivers. Although the caregivers were referred multiple times from one facility to another, they were not disappointed with such process. Concerns of extra expenditure were raised by the caregivers during the interview. However, apart from the barriers, we have identified a number of positive notations in this study. The findings are mutual trust among the healthcare professionals, sufficiently good level of communications between the service providers and a positive doctor–patient relationship. Fulfillment of treatment was also acknowledged by the caregivers interviewed in this study.

## Discussion

The national guidelines for facility-based management of SAM have been introduced to ensure proper inpatient therapeutic care of severely malnourished children^(16)^. However, the results of this study indicate that even after more than a decade of adopting the guidelines the facilities are not yet completely prepared to deliver services to the children with SAM at an optimum level. It also suggests that recommendations of national guidelines are not maintained as intended in the facilities. This finding is consistent with previous studies demonstrating poor quality of child care in public hospitals of the country^(28)^. A recent study in Bangladesh exhibited that preparedness was poor for management of sick under-five children in primary health care facilities^(29)^. The unpreparedness of facilities for the management of SAM underscores not only building up the community-based management of acute malnutrition programme but also improvement in inpatient management of SAM^(30)^. The study respondents also emphasised reinforcing the community-based management of acute malnutrition programme in the community. Implementation of growth monitoring and promotion activities and identification of children with acute malnutrition in the community using mid-upper arm circumference screening may help to improve nutritional outcomes in young children^(31)^. In addition, effective counseling, appropriate social safety net programme and provision of therapeutic food on a targeted basis in community level would help to reduce hospitalisation as well as adverse consequences of acute malnutrition in children^(32)^. However, the facility-based services are still important for detection and management of SAM in under-five children^(33)^. Thus, efforts need to be taken to improve quality of services in the hospital settings. The WHO has emphasised on infrastructural development, supply of essential drugs and equipment, skilled health workforce and guideline-based management for improving the quality of care in the facilities^(34,35)^. To that end, provision of essential medicines, a stock of necessary equipment and supplies, development of existing infrastructure and appropriate training of healthcare staff may help to overcome the gaps in relation to facility preparedness^(29,36)^.

We have identified a combination of health system and contextual barriers that inhibited the facility-based management of SAM. The health system barriers include inadequate manpower, rapid turnover of staff, increased workload, insufficient training of the service providers and lack of adherence to the management protocol as recommended in the guidelines. Bangladesh is in a critical stage for lack of trained health workforce including doctors and nurses^(37,38)^. Shortage of skilled service providers and rapid turnover of healthcare staff immensely affect the quality of care^(39)^. It also results in high workload pressure of existing staff in the facilities. Increased workload of health workforce has already been reported in the government hospitals of Bangladesh^(37)^. Hence, it is recommended by the WHO to increase proportion of healthcare staff and adopt flexible health workforce planning to reduce the workload of physicians and nurses in the public healthcare facilities^(35)^. We have reported a low coverage for training of the healthcare providers on the facility-based management of SAM. Perhaps, inadequate fund and opportunities for skill enhancement in remote areas are primarily responsible for this problem. It is also possible that most of the healthcare staff missed the training programme due to rapid turnover. In addition, insufficient training programmes during the period of COVID-19 pandemic can be another possible cause for lack of training. Further studies are required to elucidate the reasons for inadequate training of healthcare professionals. However, the inadequate training could be a probable reason for lack of adherence to the management protocol as recommended in the national guidelines. Earlier works showed that supervised training improves perception as well as adherence to any standard protocols or guidelines^(40–42)^. The WHO has also endorsed for investment in skill development of healthcare providers in order to improve the quality of hospital services^(43)^. Appropriate training with regulated supervision may improve the quality of care and ensure adherence of the healthcare providers to the management guidelines^(28)^.

The facility barriers observed in this study are unavailability of necessary medical supplies, required equipment and essential medicines in the facilities, insufficient space and beds in the SAM corner, unavailability of resources for laboratory investigations and difficulties in managing food for admitted children. Presence of functional anthropometric tools is essential for screening of undernutrition and regular assessment of nutritional status of the sick children^(29)^. Lack of anthropometric apparatus in the hospital settings may lead to inaccurate diagnosis of children with undernutrition and thwart the appropriate management of malnutrition^(44)^. Misdiagnosis of SAM in the facilities may also deprive the malnourished children from receiving specialised care designed for this vulnerable group of patients^(45)^. Unavailability was also reported for glucometer, thermometer, paediatric sphygmomanometer and necessary medical supplies. Children with SAM are prone to develop hypoglycaemia that may further increase the risk of mortality among critically ill children^(46)^. It is, therefore, imperative to monitor blood glucose level of severely malnourished child at a regular interval. Glucometer is a widely used point-of-care-testing device for bedside monitoring of blood glucose levels^(47)^. However, we found that two of the hospitals did not have a glucometer in the paediatric ward. Lack of glucometer in the hospitals is a potential barrier to ascertain the protocol-based management. Supply of glucose to treat hypoglycaemia is similarly important. It was discovered from the interviews and observations that supply of glucose was scarce in the facilities. Similar pattern was identified for essential medicines including broad-spectrum antibiotics, micronutrient supplements, ReSoMal, F-75 and F-100.

The national guidelines suggest immediate administration of broad-spectrum antibiotics to the hospitalised children with SAM^(16)^. In addition, correction of micronutrient deficiency in children with SAM is recommended as it reduces the risk of diarrhoea, respiratory infections and ocular manifestations^(48,49)^. It is also evident that administration of micronutrient supplements contributes to better health outcome in malnourished children^(49)^. ReSoMal is the first choice of fluid to correct dehydration in severely malnourished children^(16)^. However, we observed no supply of ReSoMal in all the selected facilities. Instead, glucose-based oral saline was used for the management of dehydration in the hospitals. Oral saline is high in Na that may cause sodium-overload in severely malnourished children^(50)^. Most of the physicians interviewed in this study recommended a constant supply of ReSoMal, F-75 and F-100 in the paediatric units. F-75 and F-100 are important therapeutic products which are developed for management of malnourished children. In our study, we found that half of the facilities (*n* 3) did not have the supply of F-75 and F-100. Insufficient supply of medicines and foods in the public facilities results in out-of-pocket payment of caregivers owing to purchasing the products from outside sources^(51)^. Hence, there remains a chance of missed dosage of medicines for children from poor families. Continuous supply of required drugs at the facilities could reduce the cost of caregivers and ensure the proper treatment of children with SAM in the facilities.

Although we have observed growth charts and record keeping register books in all the selected facilities, the printed version of the guidelines for facility-based management of SAM was unavailable in four of the hospitals. A study conducted in India showed that absence of institutional guidelines for clinical management led to confusion and different treatment practices among the healthcare professionals^(52)^. We also observed lack of adherence of healthcare providers towards management protocol as recommended in the guidelines. In Bangladesh, the National Nutrition Services has been implemented to strengthen the infrastructure of public healthcare facilities for management of malnourished children^(29)^. Nevertheless, we observed infrastructural inadequacy with insufficient space and limited beds for children with SAM in the facilities. Increasing number of beds and spacious corners dedicated for children with SAM could play an important role in improving the outcome of facility-based management of SAM. In addition, special attention needs to be given on cleanliness of the wards, supply of adequate drinking water and arrangement of resting place for caregivers.

In our assessment, caregivers’ reluctance to complete the treatment regimen and their insufficient knowledge regarding malnutrition as well as proper feeding were the principal contextual barriers. In addition, increased number of attendants affecting services and poverty of parents were identified as key factors in hindering the facility-based management of SAM. Well-constructed counselling sessions by trained and efficient healthcare providers would improve the knowledge of the caregivers. They should also be motivated to translate the acquired knowledge into actions. Prior evidence suggests that caregivers may not adopt the practices even after education sessions by healthcare staff in the facilities^(53)^. Therefore, multiple sessions on nutrition and health education using behaviour change and communication materials are recommended to overcome this issue. Regulatory measures should be taken to control the number of attendants in the wards. Increasing supply of medicines and ensuring the laboratory investigations in the facilities could address the issue of poverty to some extent.

### Strengths and limitations

To the best of our knowledge, this is the first study to evaluate the readiness of public healthcare facilities and investigate the barriers to the facility-based management of severely malnourished children in Bangladesh. The strength of the study includes inclusion of six public healthcare settings from two administrative divisions of the country. In addition, interview of healthcare providers and subject experts enables us to excavate several structural barriers through the application of qualitative methodology. Triangulation of interview findings with data obtained from observation was also strengthened our results. However, there are several limitations as well. Lack of generalisability and cross-sectional nature of the study may have limited the robustness of the findings. Nevertheless, saturated themes emerged from the interviews. Moreover, we could interview only four caregivers in this study which restricted us to evaluate the barriers from the perspective of demand side. Radiology and sonography were not included in the observation checklist which is another limitation of the study.

## Conclusions

Our findings suggest that facilities were not completely ready to deliver inpatient services to the children with SAM. A number of health system and facility-related barriers were identified which are curbing the facility-based management of severely malnourished children. Recruitment of skilled healthcare providers, proper on job training and adequate supply of necessary logistics may alleviate these challenges. Besides, proper functioning of SAM corners, arrangement of resources for laboratory investigations and allocation of dedicated staff for management of SAM would improve the management. Proper counselling of caregivers may reduce the obstacles from demand side. Trained healthcare staff to be assigned for the purpose of multiple counselling sessions. In addition, strengthening of community-based management of acute malnutrition programme, implementation of growth monitoring and promotion and recruitment of field level staffs are recommended to avert the unfavorable consequences of acute malnutrition in children. Moreover, there needs policy efforts to develop feasible interventions in order to reduce the barriers and ensure preparedness of the facilities for effective service delivery.
